# Verification of the Relationship between Redox Regulation of Thioredoxin Target Proteins and Their Proximity to Thylakoid Membranes

**DOI:** 10.3390/antiox11040773

**Published:** 2022-04-13

**Authors:** Yuka Fukushi, Yuichi Yokochi, Ken-ichi Wakabayashi, Keisuke Yoshida, Toru Hisabori

**Affiliations:** 1 Laboratory for Chemistry and Life Science, Institute of Innovative Research, Tokyo Institute of Technology, Yokohama 226-8503, Japan; fukushi.y.aa@m.titech.ac.jp (Y.F.); Yuichi.Yokochi@kaneka.co.jp (Y.Y.); wakaba@res.titech.ac.jp (K.-i.W.); yoshida.k.ao@m.titech.ac.jp (K.Y.); 2 School of Life Science and Technology, Tokyo Institute of Technology, Yokohama 226-8503, Japan; 3 Products Technology Team, Supplement Strategic Unit, Pharma & Supplemental Nutrition Solutions Vehicle, KANEKA Corporation, Takasago 676-8688, Japan

**Keywords:** redox regulation, thioredoxin, chloroplast, thioredoxin target protein

## Abstract

Thioredoxin (Trx) is a key protein of the redox regulation system in chloroplasts, where it modulates various enzyme activities. Upon light irradiation, Trx reduces the disulfide bonds of Trx target proteins (thereby turning on their activities) using reducing equivalents obtained from the photosynthetic electron transport chain. This reduction process involves a differential response, i.e., some Trx target proteins in the stroma respond slowly to the change in redox condition caused by light/dark changes, while the ATP synthase γ subunit (CF_1_-γ) located on the surface of thylakoid membrane responds with high sensitivity. The factors that determine this difference in redox kinetics are not yet known, although here, we hypothesize that it is due to each protein’s localization in the chloroplast, i.e., the reducing equivalents generated under light conditions can be transferred more efficiently to the proteins on thylakoid membrane than to stromal proteins. To explore this possibility, we anchored SBPase, one of the stromal Trx target proteins, to the thylakoid membrane in *Arabidopsis thaliana*. Analyses of the redox behaviors of the anchored and unanchored proteins showed no significant difference in their reduction kinetics, implying that protein sensitivity to redox regulation is determined by other factors.

## 1. Introduction

The redox regulation system in chloroplasts is responsible for the light-responsive control of various chloroplast proteins. Thioredoxin (Trx) is a small and ubiquitous protein containing two reactive cysteines, which, in this system, reduces the disulfide bonds of Trx target proteins via a dithiol–disulfide exchange reaction. When the photosynthetic electron transport chain is driven by light, part of the reducing equivalents is transferred to ferredoxin (Fd) and then to Trx via ferredoxin-thioredoxin reductase (FTR) [1,2]. Trx then transfers the reducing equivalents to the target proteins, a reaction that generally turns on their activities. Whole-genome analysis of *Arabidopsis thaliana* has identified five Trx subtypes (Trx-*f,* Trx-*m*, Trx-*x*, Trx-*y*, and Trx-*z*) in chloroplasts, each of which has different target selectivity and expression levels [3,4,5,6,7]. Moreover, Trx regulates a variety of chloroplast proteins involved in the regulation of diverse chloroplast functions [8]. For example, several Calvin–Benson cycle enzymes such as fructose-1, 6-bisphosphatase (FBPase) and sedoheptulose-1, 7-bisphosphatase (SBPase), and the chloroplast-localized ATP synthase γ subunit (CF_1_-γ) are well-known for their redox-responsive properties [9,10,11,12,13,14]. Thus, Trx plays a key role in the modulation of chloroplast function through the redox regulation of various targets in response to changes in the light environment.

In general, chloroplast proteins undergo light-dependent redox regulation to coordinately control photosynthetic and metabolic reactions under light conditions. While stroma-localized Trx target proteins, such as FBPase and SBPase, are gradually reduced with changes in light intensity and light exposure time in vivo, CF_1_-γ is reduced immediately just after light exposure [11,12,13]. In addition, the response of rubisco activase (RCA), localized mainly to stroma and partly to the thylakoid membrane, is in-between those of stromal Trx target proteins and CF_1_-γ [13,15]. Currently, the factors that determine the differential redox responses of Trx target proteins are unknown. Furthermore, the mechanism underlying the rapid redox kinetics of CF_1_-γ and its physiological significance have not yet been elucidated.

We hypothesized that the differences in redox responses of Trx target proteins are due to differences in protein localization in the chloroplast. The localization of CF_1_-γ on the thylakoid membrane as a subunit of chloroplast ATP synthase may lead to its rapid redox behavior, because the reducing equivalents from the photosynthetic machinery in thylakoid membrane are transferred more efficiently to thylakoid membrane-bound CF_1_-γ than to stromal Trx target proteins.

Our aim was to gain insight into the mechanism underlying highly sensitive redox regulations by verifying the relationship between the subchloroplast localization of Trx target proteins and their reduction kinetics. To accomplish this task, we generated an *A. thaliana* mutant that expresses a membrane-bound SBPase consisting of a thylakoid transmembrane domain (TM domain) fused to SBPase, a stromal Trx target protein. We then analyzed the reduction profiles of this membrane-bound protein. The wild-type SBPase is a dimer protein with the slowest redox behavior among previously analyzed Trx target proteins [13]. Therefore, it is an ideal protein to analyze the effect of thylakoid membrane localization on protein reduction kinetics. The TM domain of thylakoid membrane-localized ascorbate peroxidase (APX) [16] was fused to SBPase because APX is located near photosystem I and its C-terminus TM domain is not involved in its enzymatic activity [17,18,19]. Our results show that changing the localization of SBPase within the chloroplast does not change its redox profile, suggesting that the sensitivity of CF_1_-γ to redox regulation is not dictated by its localization but by other factors.

## 2. Materials and Methods

### 2.1. Plant Materials and Growth Conditions

Wild-type *A. thaliana* (Col-0) was used as a control and designated as the “WT” strain. The WT strain served as background for generating plants overexpressing SBPase fused to the TM domain (SBPase-TM_APX_), and these were designated “OE-SBPase-TM_APX_”. By targeting the *Sal*I–*Sac*I fragments, we amplified the sequence encoding the TM domain region (Glu369–Phe426) from cDNA encoding the APX gene (*At1g77490*). The amplified PCR fragments were then cloned into the pBlueScript vector (Agilent) and then the TM_APX_ fragments were amplified as *Sal*I–*Sac*I fragments by PCR. We used SBPase gene cDNA (*At3g55800*) to amplify the sequence encoding the chloroplast transit signal peptide of SBPase (Met1–Lys59). The amplified genes were fused by over-lap PCR [20] with another DNA fragment encoding the mature region of SBPase (Ala60–Glu396) and the Hexa-His-tag was added to its C-terminus. This fragment was amplified from previously modified pET-23c SBPase constructs [21]. The full-length SBPase included a chloroplast transit signal sequence and Hexa-His-tag fused fragments cloned into the pRI 201-AN vector (Takara) with the aforementioned TM_APX_ fragments. The sequences of the primers used for these experiments are shown in Table S1. To construct an SBPase-TM_APX_ overexpressing plant, OE-SBPase-TM_APX,_ plants were transformed with the aforementioned pRI 201-AN construct with SBPase-TM_APX_ encoding region by the agrobacterium-mediated floral dip method [22]. Plants were grown in soil under a 16 h light (50–60 μmol photons m^−2^ s^−1^)/8 h dark cycle at 22 °C for 4 weeks.

### 2.2. Protein Extraction from Plant Leaves

Plant leaves were frozen in liquid nitrogen and homogenized with leaf protein extraction solution (2% (*w*/*v*) SDS, 62.5 mM Tris-HCl (pH 6.8), and 7.5% (*v*/*v*) glycerol) and the homogenate was heat-treated at 95 °C for 5 min and then centrifuged at 20,400× *g* for 15 min. The concentration of leaf proteins in the supernatant was determined using the BCA protein assay kit (Pierce). To the supernatant, 1/3 of the supernatant volume of SDS sample buffer (8% (*w*/*v*) SDS, 250 mM Tris-HCl (pH 6.8), 30% (*v*/*v*) glycerol, and 0.04% (*w*/*v*) bromophenol blue) containing 20% (*v*/*v*) 2-mercaptoethanol was added and then the mixture was subjected to SDS-polyacrylamide gel electrophoresis (PAGE). Proteins were transferred to a PVDF membrane by Western blotting.

### 2.3. Measurements of Plant Growth and Chlorophyll Content

The aboveground fresh weight (FW) of plants was measured. The chlorophyll content in rosette leaves was calculated as the sum of the contents of chlorophyll *a* and *b* after extraction of the chlorophyll with 80% (*v*/*v*) acetone according to previously published methods [23].

### 2.4. Isolation and Sub-Fractionation of Chloroplasts

Chloroplast stroma and thylakoid membrane fractions were obtained by collecting intact chloroplasts from plant leaves and disrupting them by osmotic stress as previously described [24].

### 2.5. Determination of Light-Dependent Protein Redox State In Vivo

Plants were dark-adapted for 8 h and then irradiated at 650–750 μmol photons m^−2^ s^−1^. The plant leaves were then harvested at the indicated times and frozen in liquid nitrogen. The redox states of the proteins in plant leaves were determined according to previously described methods [12]. To distinguish between the reduced band of endogenous SBPase and the oxidized band of SBPase-TM_APX_ in OE-SBPase-TM_APX_ plants, we used the Penta·His antibody (BSA-free, Qiagen) to detect SBPase-TM_APX_. The antibodies against SBPase and CF_1_-γ were prepared as described previously [12,21].

## 3. Results and Discussion

### 3.1. Generation of Plants Expressing Thylakoid Membrane-Anchored SBPase

**Figure 1:**
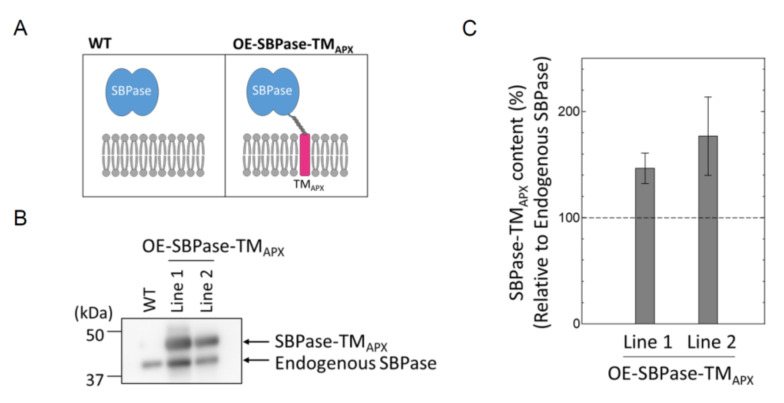
Generation of *A. thaliana* mutants expressing membrane-anchored SBPase. (**A**) Construction of SBPase-TM_APX_. The blue ovals represent SBPase and the magenta cylinder represents TM_APX_. The gray chain represents the Hexa-His-tag linker connecting SBPase to TM_APX_. (**B**) SBPase-TM_APX_ expression in two independent lines was confirmed by Western blotting. (**C**) The relative expression level of SBPase-TM_APX_ in OE-SBPase-TM_APX_ plant was calculated from the signal intensities shown in (**B**). The expression level of endogenous SBPase in an OE-SBPase-TM_APX_ plant was defined to 100%. Each value represents the mean ± SD (*n* = 3).

**Figure 2:**
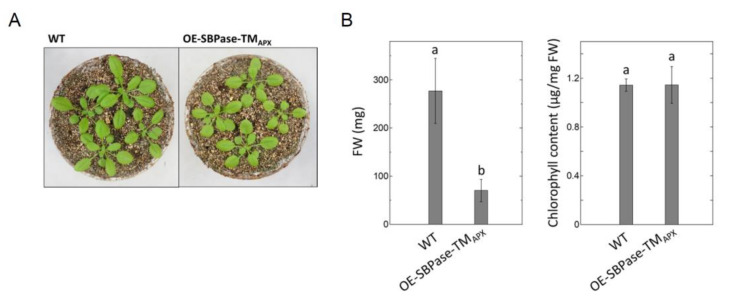
Phenotypes of mutant plants expressing membrane-anchored SBPase. (**A**) Four-week-old OE-SBPase-TM_APX_ plants. (**B**) Fresh weight (FW) and chlorophyll content of OE-SBPase-TM_APX_ plant. Each value represents the mean ± SD (*n* = 4). Different letters indicate significant differences among plant lines (*p* < 0.05; one-way ANOVA and Tukey’s honestly significant difference).

To evaluate the effect of subchloroplast localization on the reduction kinetics of Trx target proteins, we generated two membrane-bound, SBPase-overexpressing mutant lines of *A. thaliana*. In both mutant plants (designated OE-SBPase-TM_APX_), the TM domain of the membrane-bound APX was fused to SBPase. Because the thylakoid-bound APX, like CF_o_CF_1_, localizes mainly to stroma thylakoids [17,25], we expected that the redox behavior of SBPase-TM_APX_ would be similar to that of CF_1_-γ. Then we confirmed that the protein (designated SBPase-TM_APX_) was definitely anchored to the thylakoid membrane (Figure 1A). The expression of SBPase-TM_APX_ in the mutant plants was confirmed by Western blotting using antibodies against SBPase (Figure 1B). The expression levels of SBPase-TM_APX_ in both OE-SBPase-TM_APX_ plant lines were about 150–200% of endogenous SBPase (Figure 1C). We used Line 1 of the OE-SBPase-TM_APX_ plants for subsequent analyses. Figure 2 shows the growth phenotype, FW and chlorophyll content of this OE-SBPase-TM_APX_ plants. Compared to WT, the growth of OE-SBPase-TM_APX_ plants was stunted and its FW was lower; however, its chlorophyll content and major Trx content (data not shown) did not differ significantly from WT plants.

**Figure 3:**
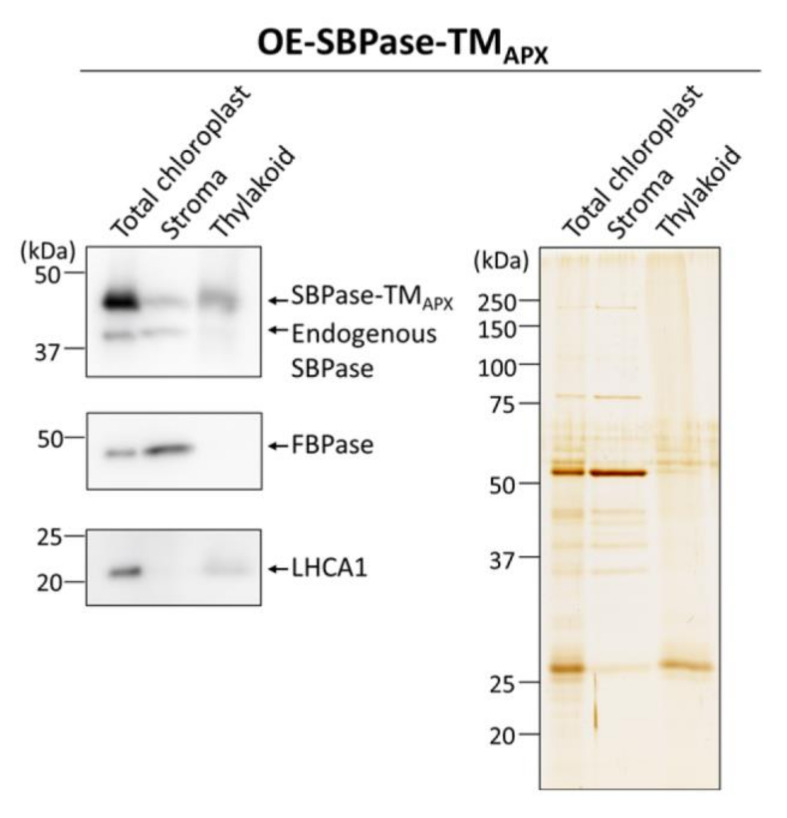
Localization of the membrane-anchored SBPase in chloroplasts. Proteins from stroma and thylakoid membrane fractions of leaves in OE-SBPase-TM_APX_ plants were prepared for immunoblot analysis. The same amounts of proteins were loaded into each lane as shown in an SDS-PAGE gel in the right panel (silver-stained). Antibodies against SBPase was used to detect the endogenous SBPase and SBPase-TM_APX_. FBPase and light-harvesting complex protein LHCA1 were detected as markers of stroma and thylakoid membrane fractions, respectively, using their specific antibodies.

To determine whether SBPase-TM_APX_ is anchored to the chloroplast thylakoid membrane in mutant plants, we isolated the intact chloroplasts from leaves and then obtained stroma and thylakoid membrane fractions. Immunoblot analyses using antibodies against SBPase showed that SBPase-TM_APX_ is mainly localized to the thylakoid membrane in OE-SBPase-TM_APX_ plants (Figure 3). To anchor SBPase to thylakoid membrane, we used the TM domain of APX as described above. Thus, since we have confirmed that SBPase-TM_APX_ localized to the thylakoid membrane (Figure 1A), we expect the localization of this enzyme on the thylakoid membrane to be similar to that of CF_1_-γ.

### 3.2. In Vivo Redox Responses of Thylakoid Membrane-Anchored SBPase 

To evaluate the effect of subchloroplast localization on the reduction kinetics of Trx target proteins, we analyzed the reduction kinetics of SBPase-TM_APX_ in vivo. As a reference, we also analyzed the reduction kinetics of thylakoid membrane-bound CF_1_-γ. The reduction rate was measured by irradiating plant leaves at high-intensity light (650–750 μmol photons m^−2^ s^−1^) after dark adaptation and then observing the changes in the redox state of proteins by thiol modification as described previously [12].

**Figure 4:**
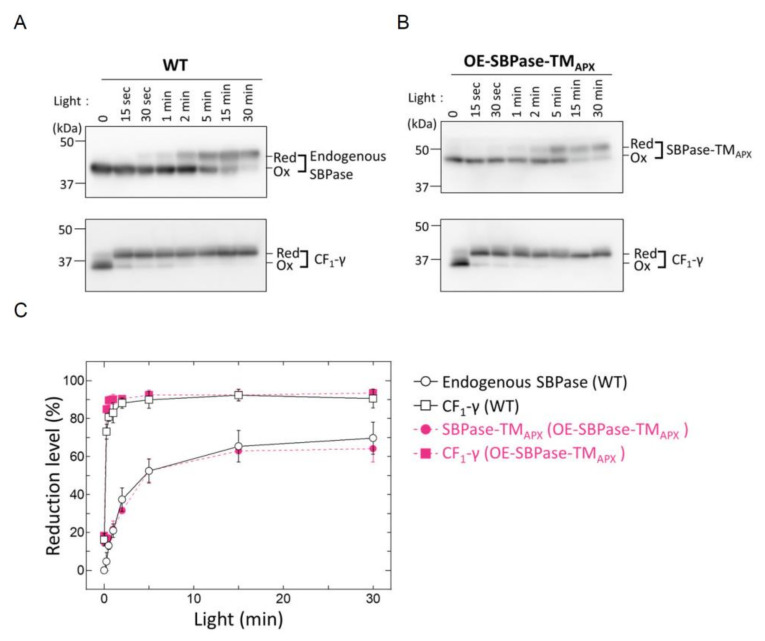
In vivo redox responses of membrane-anchored SBPase. Dark-adapted plants were placed under light conditions (650–750 µmol photons m^−2^ s^−1^) for the specified time period and then leaves were frozen in liquid nitrogen. The redox states of endogenous SBPase, SBPase-TM_APX_ and CF_1_-γ were determined by the method previously described in [12]. Antibodies against SBPase and CF_1_-γ were used to detect the endogenous proteins, while the Penta·His-tag antibody was used to detect SBPase-TM_APX_, thus providing a clear distinction between the reduced band of endogenous SBPase and the oxidized band of SBPase-TM_APX_ in OE-SBPase-TM_APX_ plants. (**A**) The redox state of endogenous SBPase and CF_1_-γ in WT plants. (**B**) The redox state of SBPase-TM_APX_ and CF_1_-γ in OE-SBPase-TM_APX_ plants. (**C**) The reduction level of SBPase and CF_1_-γ is based on the signal intensities shown in (**A**,**B**). The reduction level was calculated as the ratio of the reduced form to the total amount of reduced and oxidized forms. Each value represents the mean ± SD (*n* = 3). Red, reduced form; Ox, oxidized form.

The reduction profiles in Figure 4 show a gradual reduction of both SBPase-TM_APX_ in OE-SBPase-TM_APX_ plants and endogenous SBPase in WT plants through light exposure time. In contrast, CF_1_-γ rapidly reached the maximum reduction level (about 90%) at approximately 120 s after light irradiation of OE-SBPase-TM_APX_ and WT plants. Thus, thylakoid membrane-bound and stroma-localized endogenous SBPases shared the same reduction kinetics, which was distinct from the rapid reduction response of CF_1_-γ.

CF_1_-γ is an abundant protein in the chloroplast, nevertheless, it is reduced rapidly even under low light conditions [13,21]. In contrast, the expression level of SBPase-TM_APX_ was about 150–200% of endogenous SBPase (Figure 1C), but the reduction rates of both proteins were almost equivalent (Figure 4). These results imply that the turnover rate of Trx (the protein responsible for transferring reducing equivalents to these target proteins) is sufficiently rapid for it to reduce all target proteins in chloroplast.

## 4. Conclusions

Based on our results, we conclude subchloroplast localization does not affect the reduction kinetics of SBPase. In chloroplasts, target proteins are continuously oxidized, even under light irradiation conditions [11], and the balance between the reduction and oxidation processes should be an important factor in determining the reduction kinetics. Another possible factor affecting the reduction kinetics is the different affinities between Trx and each Trx target protein, which are based on differences in surface electrostatic potential. Studies suggest that changes in surface electrostatic charge around the reactive cysteine pair of CF_1_-γ causes changes in Trx-dependent redox properties [26,27,28].

In addition, NADPH-thioredoxin reductase C (NTRC) has been recently proposed as another factor responsible for the rapid reduction of CF_1_-γ in addition to Trx [29,30]. NTRC reduces the target proteins using NADPH as a source of reducing equivalents [31]. Overall, these studies indicate that, rather than subchloroplast localization, the sensitivity to redox regulation of Trx target proteins may involve other complex mechanisms.

## Data Availability

Data is contained within the article.

## Supplementary Materials

The following supporting information can be downloaded at: https://www.mdpi.com/article/10.3390/antiox11040773/s1, Table S1: Primers used in this study.
