# Supplementary material for: Verification of the Relationship between Redox Regulation of Thioredoxin Target Proteins and Their Proximity to Thylakoid Membranes
Source: Antioxidants (Basel). 2022 Apr 13;11(4):773. doi: 10.3390/antiox11040773 (PMC9032623; doi:10.3390/antiox11040773)
Supplement: Supplementary file 1 [file antioxidants-11-00773-s001.zip › antioxidants-1638113-supplementary.pdf]

Supplementary Materials

Table S1. Primers used in this study.

**Name**	**Sequence (5′ to 3′)**
TM_{APX}_F[a]	AAAGTCGACGAGCTTTCGGATTCGATGAAAAAG
TM_{APX}_R[a]	ATCGAGCTCTTAGAAACCAGAGAAATCGGAGTTG
transit-SBPase_F[b,c]	TGATACATATGGAGACCAGCATCGC
transit-SBPase_R[b]	CACCATTGCTCTTAGCTTTTG
pET_SBPase_R[c]	ATTGTCGACGTGATGATGGTGGTGGTGCTCGAGAG

a: Primers used for TM_{APX} fragment preparation. The underlined parts indicate the restriction enzyme recognition sites.

b: Primers used for the preparation of SBPase fragment fused with chloroplast transit signal peptides.

c: A primer used for the overlap extension PCR.
